# Association of Upper Trapezius Muscle Status With Pain and Disability in Patients With Chronic Nonspecific Neck Pain: A Cross‐Sectional Study

**DOI:** 10.1155/prm/8174866

**Published:** 2026-10-29

**Authors:** Jialin Yang, Hanyu Zou, Fangfang Xie, Chong Guan, Jianwen Ma, Juntao Chen, Fei Yao

**Affiliations:** ^1^ School of Acupuncture-Moxibustion and Tuina Shanghai University of Traditional Chinese Medicine Shanghai China shutcm.edu.cn; ^2^ Department of Tuina Shanghai Municipal Hospital of Traditional Chinese Medicine Shanghai China szy.sh.cn; ^3^ Department of Tuina Shuguang Hospital Affiliated to Shanghai University of Traditional Chinese Medicine Shanghai China shutcm.edu.cn

**Keywords:** chronic nonspecific neck pain, cross-sectional study, disability, pain, pressure pain threshold, tissue hardness, upper trapezius

## Abstract

**Background:**

Chronic nonspecific neck pain (CNSNP) is a leading cause of disability worldwide. Alterations in upper trapezius tissue hardness and pain sensitivity may be closely associated with pain intensity and disability. This study aimed to investigate the associations of upper trapezius tissue hardness and pressure pain threshold (PPT) with pain intensity and neck disability in patients with CNSNP.

**Methods:**

This single‐center cross‐sectional study enrolled 426 patients with CNSNP (244 women and 182 men; mean age, 44.22 ± 8.84 years) at the Health Examination Center of Shuguang Hospital Affiliated to Shanghai University of Traditional Chinese Medicine between August 2024 and August 2025. Bilateral upper trapezius tissue hardness and PPT were measured using the OE220 outcome evaluation system (ITO, Japan). Pain intensity was assessed with a composite visual analog scale (VAS) score, and neck disability with the neck disability index (NDI). Spearman correlation analysis and linear regression models were used to evaluate the associations among variables, followed by prespecified subgroup analyses to explore the heterogeneity of these associations across demographic and clinical subgroups.

**Results:**

Spearman correlation analysis showed that mean hardness was positively correlated with VAS (*r* = 0.328, *p* < 0.001) and NDI (*r* = 0.173, *p* < 0.001), whereas mean PPT was negatively correlated with VAS (*r* = −0.307, *p* < 0.001) and NDI (*r* = −0.121, *p* < 0.05). Multiple linear regression analysis showed that, after full adjustment for confounding factors, mean hardness remained positively associated with VAS (*B* = 0.071, *β* = 0.268, 95% CI: 0.051∼0.091, *p* < 0.001), while mean PPT was negatively associated with VAS (*B* = −0.187, *β* = −0.113, 95% CI: −0.316∼−0.058, *p* = 0.005); the adjusted model explained 40.8% of the variance. Regarding the NDI, only mean hardness was significantly and positively associated with it (*B* = 0.180, *β* = 0.160, 95% CI: 0.075∼0.286, *p* < 0.001). In prespecified subgroup analyses, formal interaction tests showed no statistically significant effect modification for either outcome (all *p* for interaction > 0.05); the smallest *p* value, for the hardness‐by‐work‐type interaction on the NDI, was 0.122, and this exploratory observation was not regarded as evidence of effect modification.

**Conclusion:**

In patients with CNSNP, increased tissue hardness and elevated local pain sensitivity of the upper trapezius are closely associated with pain intensity, and tissue hardness is a relevant factor associated with cervical dysfunction. These findings suggest that objective upper trapezius muscle status indicators may provide complementary descriptive information in the assessment of CNSNP, although their incremental clinical value beyond conventional assessment has not been established; longitudinal studies are needed to determine whether these indicators can guide treatment or predict outcomes.

## 1. Introduction

Neck pain is a leading cause of disability worldwide, with a lifetime prevalence as high as 71% and an annual prevalence of approximately 30%–50% [1, 2]. Among all types of neck pain, chronic nonspecific neck pain (CNSNP) is the most common, accounting for more than 60% of patients with neck pain. CNSNP is characterized by neck pain lasting longer than 3 months without an identifiable pathological or neurological explanation [3]. It not only leads to physical limitation and reduced quality of life but also imposes substantial medical expenditure and socioeconomic burden, and has become a major public health concern [4].

In recent years, research on the pathogenesis of CNSNP has increasingly focused on abnormalities of cervical muscle function. Once specific pathologies have been excluded, structural and functional alterations of the cervical muscles are considered a core component in the development and maintenance of chronic pain [5, 6]. As a key superficial muscle that maintains static cervical posture and dynamic stability, the upper trapezius is particularly vulnerable to functional impairment under prolonged postural load and has therefore become a critical target in CNSNP research [6, 7]. Previous studies have provided preliminary evidence of altered upper trapezius muscle status in patients with CNSNP. Using shear‐wave elastography, Taş et al. found that shear‐wave velocity and shear modulus of the upper trapezius were significantly higher in patients with chronic neck pain than in healthy individuals, indicating a pathological increase in muscle stiffness [8]. Leong et al. further confirmed that the stiffness of the upper trapezius, levator scapulae, and sternocleidomastoid was higher in patients with CNSNP than in asymptomatic individuals [9]. Pressure pain threshold (PPT), an indicator of local pressure pain sensitivity, has also been shown to be abnormal in several studies: PPT over the upper trapezius is significantly lower in patients with CNSNP than in healthy individuals, suggesting that local hyperalgesia is an important feature of CNSNP [10–12]. Further evidence indicates that upper trapezius muscle mechanical properties and PPT are associated with pain severity and disability [13, 14] and that interventions targeting this muscle, such as extracorporeal shock wave therapy, neuromuscular inhibition techniques, and stretching exercises, have been reported to reduce muscle stiffness, modulate PPT, and relieve pain and improve function. These observations suggest that muscle mechanical properties and PPT may be relevant factors linking muscle structural and functional abnormalities to pain perception [15–17].

Despite this progress, the evidence linking muscle status to pain and disability remains inconsistent. Some studies have reported associations between muscle mechanical properties or pressure pain sensitivity and symptom severity, whereas others have found weak or no associations, and the findings appear to vary by muscle and by measurement technique. Earlier studies have also generally involved relatively small samples, and many did not measure tissue hardness and pressure pain sensitivity together or adjust sufficiently for demographic, socioeconomic, and lifestyle factors. As a result, it remains unclear whether altered upper trapezius tissue hardness and pressure pain sensitivity are independently associated with patients′ own pain intensity and disability. To address these issues, the present study examined a comparatively large sample of 426 patients, measured tissue hardness and PPT simultaneously at the upper trapezius, and used multivariable adjustment together with subgroup and interaction analyses to quantify these associations.

Accordingly, the present study analyzed data from 426 patients with CNSNP with two primary objectives: (1) to investigate the associations between upper trapezius muscle status (tissue hardness and PPT) and pain severity (VAS score) and neck disability (NDI score) and (2) to examine whether these associations varied across population characteristics through prespecified subgroup and interaction analyses, thereby providing evidence on the relevance of the upper trapezius in CNSNP.

## 2. Methods

### 2.1. Study Design

This single‐center, observational, cross‐sectional study was approved by the Ethics Committee of Shuguang Hospital Affiliated with Shanghai University of Traditional Chinese Medicine (approval no. 2024‐1587‐170‐01), and all participants provided written informed consent. Patients with CNSNP who visited the outpatient clinic of the Department of Rehabilitation Medicine at a tertiary hospital between August 2024 and August 2025 were consecutively enrolled. The study flowchart is presented in Figure 1.

**FIGURE 1:**
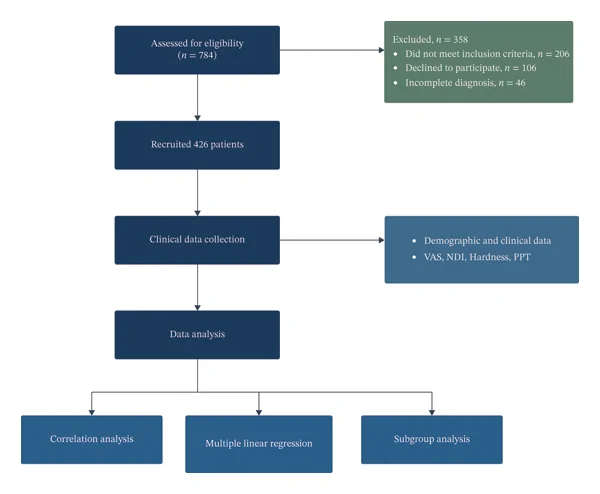
Study flowchart. VAS, visual analog scale; NDI, neck disability index; PPT, pressure pain threshold.

Inclusion criteria: (1) age 20–60 years; (2) a diagnosis of CNSNP, with neck and shoulder pain lasting more than 3 months, VAS score ≥ 3, and NDI ≥ 10; (3) imaging (cervical X‐ray or MRI) showing no definite pathological changes such as cervical fracture, tumor, infection, spinal cord compression, or severe cervical disc herniation; (4) ability to cooperate with muscle stiffness and PPT measurements and questionnaire assessment; and (5) no specific treatment for neck pain, such as manipulation, acupuncture, or physical modalities, within the previous month.

Exclusion criteria: (1) a definite history of cervical surgery or trauma; (2) a diagnosis of specific neck pain such as cervical radiculopathy, myelopathy, cervical spinal stenosis, fracture, tumor, or infection; (3) systemic inflammatory diseases such as rheumatoid arthritis or ankylosing spondylitis; (4) severe cardiovascular or cerebrovascular disease, psychiatric illness, or cognitive impairment precluding cooperation with assessment; (5) pregnancy or lactation; and (6) skin breakdown or infection at the assessment sites.

The VAS ≥ 3 and NDI ≥ 10 values were additional eligibility thresholds set for this study and were not part of the diagnostic definition of CNSNP. They were chosen to ensure that participants reported clinically meaningful pain and at least mild functional impairment, based on established VAS cut‐off points for mild pain and the standard NDI grading [18–20].

### 2.2. Assessment Measures and Instruments

#### 2.2.1. General Characteristics

A self‐administered questionnaire was used to collect demographic information (age, sex, height, weight, BMI, marital status, and education level) and lifestyle information (smoking status, alcohol consumption, type of work, and daily working hours).

#### 2.2.2. Pain Intensity

Pain intensity was assessed using the VAS. The scale consists of a 10‐cm line anchored at 0 (no pain) and 10 (worst imaginable pain). Patients marked the point on the line that corresponded to their pain, and the distance from the 0 end to the mark was measured as the VAS score [18, 21]. The VAS has well‐established reliability and validity and is one of the most widely used pain assessment tools. For the analysis, a composite pain intensity score was calculated as the average of three VAS ratings: (1) the worst pain intensity reported by the participant over the previous 4 weeks, (2) pain intensity at the time of data collection, and (3) the average pain intensity reported over the previous 4 weeks. Combining a current rating with two retrospective ratings was intended to capture both momentary and recalled pain and to reduce the influence of any single assessment, consistent with the multi‐item approach of the Brief Pain Inventory [22]. However, the Brief Pain Inventory integrates these ratings within a validated multi‐item instrument and does not by itself establish the validity of averaging current, worst, and 4‐week average pain into one equally weighted score; to our knowledge, this specific composite score has not been independently validated, which we now acknowledge explicitly as a limitation. The three individual ratings were not retained, so a component‐specific sensitivity analysis could not be performed.

#### 2.2.3. Neck Disability

Neck disability was assessed using the NDI, the most widely used functional scale for neck pain, with well‐established reliability and validity. The NDI comprises 10 items covering pain intensity, personal care, lifting, reading, headaches, concentration, work, driving, sleeping, and recreation. Each item is rated on a 0 to 5 scale, yielding a total score of 0–50, with higher scores indicating greater disability. Grading is as follows: 0 to 10, mild disability; 11 to 20, moderate; 21 to 30, severe; and 31 to 50, complete disability [19, 20].

#### 2.2.4. Upper Trapezius Tissue Hardness

Tissue hardness was measured with the OE220 outcome evaluation system (ITO, Japan), a handheld indentation device that incorporates a tissue hardness meter. The OE220 portable tissue hardness tester measures the rebound force of soft tissues by using two sensors to separately monitor the rebound force and indentation force of the tissues. It measures the rebound force of the tissues when the sum of the two forces remains constant. The ratio of the sum of the readings from the two sensors to the tissue rebound value is displayed as a percentage, which serves as the hardness value; higher values indicate harder tissue. This indentation‐based quantity differs in physical principle from the shear‐wave speed or elastic modulus obtained with shear‐wave elastography. The measurement sites were the same as those used for PPT. Each site was measured three times at 30‐s intervals, and the mean of the three measurements was used as the tissue hardness value for that site [23]. The upper trapezius measurement points were selected according to the SENIAM standards of the European Biomedical Health Research Project (https://seniam.org/) and relevant literature [23–25] (Figure 2). During measurement, the patient was seated with the neck naturally relaxed; the probe of the tissue hardness meter was held perpendicular to the muscle surface, avoiding bone or tendon, to ensure that the muscle belly was measured. All measurements were performed by a single investigator who had undergone standardized training and followed the instrument operating protocol strictly to minimize measurement error (Figure 3). This method has been shown to have good inter‐rater and test–retest reliability and can be used to assess tissue hardness in patients with neck pain. The mean of the left and right hardness values was used in the primary analysis, and left and right values were retained for side‐specific analyses.

**FIGURE 2:**
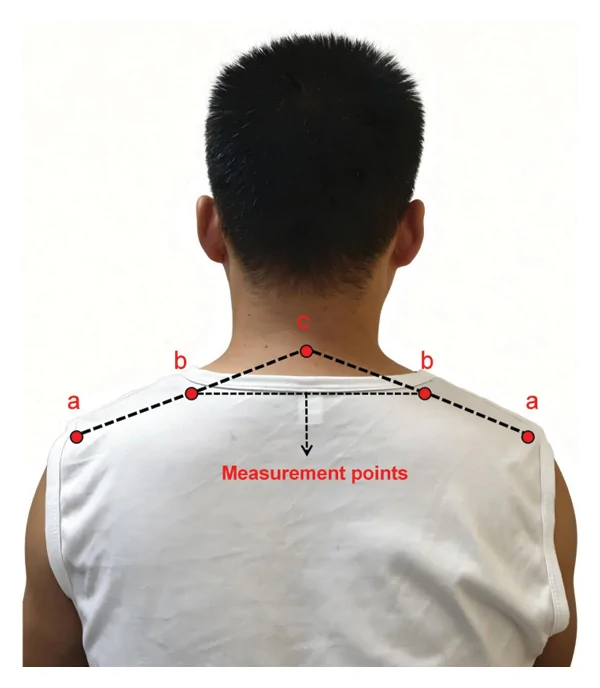
Muscle status measurement locations. (a) Acromion; (b) measurement point (midpoint between the C7 spinous process and the acromion); (c) C7 spinous process.

**FIGURE 3:**
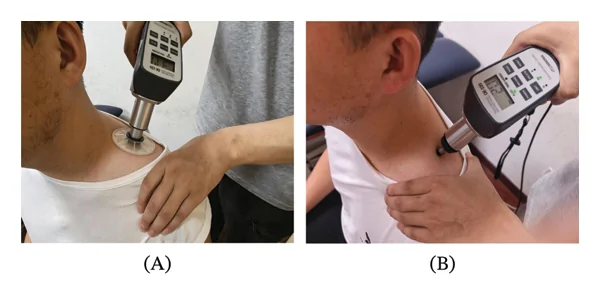
(A) Tissue hardness measurement; (B) pressure pain threshold (PPT) assessment.

#### 2.2.5. PPT

Bilateral upper trapezius PPT was measured with the OE220 outcome evaluation system (ITO, Japan). With the patient seated and the neck relaxed, the algometer probe (1‐cm^2^ contact area) was applied perpendicular to the skin surface, and pressure was increased at a rate of approximately 0.5 kg/s. Patients were instructed to report immediately when the sensation changed from pressure to pain, and the pressure at that point (kg/cm^2^) was recorded. Each point was measured three times at intervals of at least 30 s to avoid sensitization or adaptation, and the mean of the three values was used. Lower PPT values indicate greater local pain sensitivity. This method is widely used in neck pain research and has good reliability [26, 27]. The mean of the left and right PPT values was used in the primary analysis.

### 2.3. Statistical Analysis

Statistical analyses were performed with SPSS 29.0 and R 4.3.2. Normality was tested with the Shapiro–Wilk test. Normally distributed continuous variables are expressed as mean ± standard deviation and were compared between groups with the independent‐samples *t* test; non‐normally distributed variables are expressed as median (interquartile range) and were compared with the Wilcoxon rank‐sum test. Categorical variables are expressed as frequency (percentage) and were compared with the chi‐square test.

Spearman rank correlation was used to examine the associations between muscle status indicators and VAS and NDI scores. Multiple linear regression was used to assess the associations of muscle status with VAS and NDI. Covariates were selected before analysis on the basis of established clinical and causal relevance rather than statistical significance. Three nested models were constructed: Model 1 was unadjusted; Model 2 added basic demographic and anthropometric factors (age, sex, and BMI); and Model 3 additionally included socioeconomic and lifestyle factors (marital status, education level, type of work, daily working hours, smoking status, and alcohol consumption). Unstandardized regression coefficients (B), standard errors, standardized coefficients (β), 95% confidence intervals, and *p* values are reported. Regression assumptions were assessed with residual‐versus‐fitted and component‐plus‐residual plots for linearity, the Breusch–Pagan test and residual plots for homoscedasticity, Q–Q plots with the Jarque–Bera test for normality of residuals, Cook’s distance for influential observations, and the variance inflation factor (VIF) for multicollinearity. Because mild heteroscedasticity was detected for the VAS model, heteroscedasticity‐robust (HC3) standard errors were used as a sensitivity check and produced the same conclusions. Prespecified stratified analyses were performed by sex, age, BMI, marital status, education level, type of work, daily working hours, smoking status, and alcohol consumption; effect modification was evaluated with product–term interaction tests rather than by comparing significance across strata. Subgroup analyses were interpreted as exploratory given the multiple comparisons and the limited within‐stratum sample sizes. All tests were two‐sided, and *p* < 0.05 was considered statistically significant.

## 3. Results

### 3.1. Baseline Characteristics of Participants

Among 784 patients screened for CNSNP, 358 were excluded and 426 were finally included in the analysis. The mean age was 44.22 ± 8.84 years, 57.3% were women, and the mean BMI was 21.79 ± 3.38 kg/m^2^. The median VAS score was 5.0 (4.5, 5.8), and the median NDI score was 18.0 (15.0, 21.0). The median mean upper trapezius hardness was 66.0% (64.0%, 69.0%), with the right side (68.0%) higher than the left (64.0%, *p* < 0.001). The median mean PPT was 3.40 kg/cm^2^ (3.05, 3.90). Female patients had significantly higher VAS (5.1 vs. 4.8, *p* < 0.001) and NDI (19.0 vs. 17.0, *p* = 0.002) and significantly lower PPT (3.25 vs. 3.53 kg/cm^2^, *p* < 0.001) than male patients (Table 1).

**TABLE 1:** Baseline characteristics of participants (*N* = 426).

Characteristic	Overall (*N* = 426)^1^	Female (*N* = 244)^1^	Male (*N* = 182)^1^	Statistic *t/W/* *χ* ^2^	*p* value
Age (years)	44.22 ± 8.84	45.17 ± 8.69	42.95 ± 8.91	2.57	0.010^2^
Height (cm)	166.24 ± 6.65	162.19 ± 4.83	171.67 ± 4.56	−20.68	< 0.001^2^
Weight (kg)	60.29 ± 10.40	55.23 ± 8.28	67.08 ± 9.00	−13.90	< 0.001^2^
BMI (kg/m^2^)	21.79 ± 3.38	21.04 ± 3.32	22.80 ± 3.20	−5.51	< 0.001^2^
VAS	5.0 (4.5, 5.8)	5.1 (4.7, 6.0)	4.8 (4.4, 5.4)	27272.0	< 0.001^3^
NDI	18.0 (15.0, 21.0)	19.0 (15.0, 22.0)	17.0 (14.0, 21.0)	26040.5	0.002^3^
L‐UT hardness (%)	64.0 (61.0, 67.0)	64.0 (61.0, 67.0)	63.0 (61.0, 66.0)	23582.5	0.272^3^
R‐UT hardness (%)	68.0 (65.0, 72.0)	68.0 (65.0, 72.0)	66.5 (64.0, 71.0)	26090.0	0.002^3^
Mean hardness (%)	66.0 (64.0, 69.0)	66.0 (64.0, 69.5)	65.0 (63.5, 68.5)	25392.0	0.011^3^
L‐UT PPT (kg/cm^2^)	3.30 (2.80, 4.00)	3.15 (2.70, 3.80)	3.40 (2.90, 4.00)	18805.0	0.007^3^
R‐UT PPT (kg/cm^2^)	3.40 (2.90, 4.10)	3.20 (2.80, 4.00)	3.60 (3.00, 4.20)	18101.5	0.001^3^
Mean PPT (kg/cm^2^)	3.40 (3.05, 3.90)	3.25 (2.95, 3.70)	3.53 (3.15, 4.00)	17548.5	< 0.001^3^
Work duration, *n* (%)				4.10	0.043^4^
< 8 h/day	166 (39.0)	85 (34.8)	81 (44.5)		
≥ 8 h/day	260 (61.0)	159 (65.2)	101 (55.5)		
Marital status, *n* (%)				2.32	0.128^4^
Married	284 (66.7)	170 (69.7)	114 (62.6)		
Unmarried	142 (33.3)	74 (30.3)	68 (37.4)		
Smoking status, *n* (%)				142.90	< 0.001^4^
Never smoker	274 (64.3)	213 (87.3)	61 (33.5)		
Former smoker	39 (9.2)	17 (7.0)	22 (12.1)		
Current smoker	113 (26.5)	14 (5.7)	99 (54.4)		
Alcohol consumption, *n* (%)				100.16	< 0.001^4^
Yes	127 (29.8)	26 (10.7)	101 (55.5)		
No	299 (70.2)	218 (89.3)	81 (44.5)		
Type of work, *n* (%)				0.03	0.872^4^
Mental work	385 (90.4)	221 (90.6)	164 (90.1)		
Physical work	41 (9.6)	23 (9.4)	18 (9.9)		
Education level, *n* (%)				0.80	0.372^4^
College degree or higher	277 (65.0)	163 (66.8)	114 (62.6)		
High school or lower	149 (35.0)	81 (33.2)	68 (37.4)		

*Note:* L‐UT hardness, left upper trapezius tissue hardness; R‐UT hardness, right upper trapezius tissue hardness.

Abbreviations: BMI, body mass index; L‐UT PPT, left upper trapezius pressure pain threshold; Mean PPT, mean pressure pain threshold; NDI, neck disability index; R‐UT PPT, right upper trapezius pressure pain threshold; VAS, visual analog scale.

^1^Median (Q1, Q3), Mean ± SD, *n* (%).

^2^Welch two‐sample *t*‐test.

^3^Wilcoxon rank sum test.

^4^Pearson’s Chi‐squared test.

### 3.2. Correlations Between Muscle Status and Clinical Outcomes

Spearman correlation analysis showed that mean upper trapezius hardness was weakly to moderately positively correlated with VAS (*r* = 0.328, *p* < 0.001) and weakly positively correlated with NDI (*r* = 0.173, *p* < 0.001). Mean PPT was weakly to moderately negatively correlated with VAS (*r* = −0.307, *p* < 0.001) and weakly negatively correlated with NDI (*r* = −0.121, *p* < 0.05). Side‐specific analyses showed that the correlation between right upper trapezius hardness and VAS (*r* = 0.304, *p* < 0.001) was somewhat stronger than that for the left side (*r* = 0.202, *p* < 0.001), whereas the correlation coefficients for bilateral PPT with VAS were similar (left, *r* = −0.241; right, *r* = −0.252; both *p* < 0.001). For NDI, left‐sided hardness (*r* = 0.170, *p* < 0.001) and left‐sided PPT (*r* = −0.105, *p* < 0.05) were significantly correlated, whereas right‐sided hardness (*r* = 0.093, *p* > 0.05) and right‐sided PPT (*r* = −0.070, *p* > 0.05) were not (Figure 4).

**FIGURE 4:**
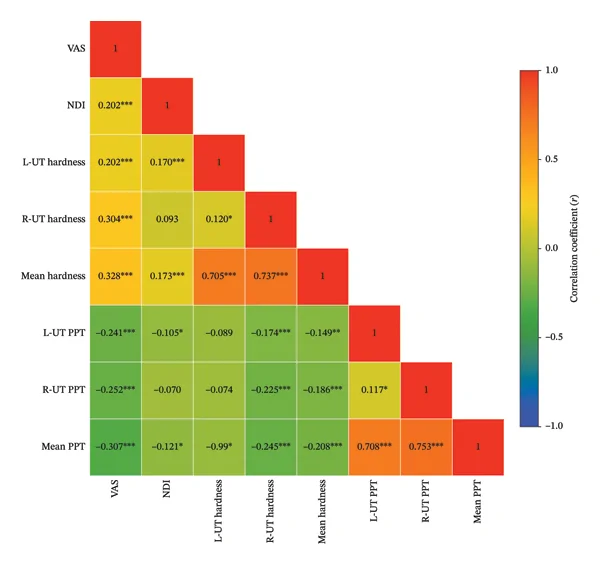
Heatmap of Spearman correlation analysis. ^∗^ *p* < 0.05, ^∗∗^ *p* < 0.01, ^∗∗∗^ *p* < 0.001. VAS, visual analog scale; NDI, neck disability index; L‐UT hardness, left upper trapezius tissue hardness; R‐UT hardness, right upper trapezius tissue hardness; L‐UT PPT, left upper trapezius pressure pain threshold; R‐UT PPT, right upper trapezius pressure pain threshold; Mean PPT, mean pressure pain threshold.

### 3.3. Regression Analysis of Upper Trapezius Status and Pain Intensity

Multiple linear regression with VAS as the dependent variable showed that in the unadjusted model (Model 1), both mean hardness (*β* = 0.324, *p* < 0.001) and mean PPT (*β* = −0.220, *p* < 0.001) were significantly associated with VAS, with an adjusted *R* ^2^ of 0.184. After adjustment for age, sex, and BMI (Model 2), the associations remained highly significant. After further adjustment for all covariates (Model 3), mean hardness remained positively associated with VAS (*B* = 0.071, *β* = 0.268, 95% CI: 0.051∼0.091, *p* < 0.001), and mean PPT was negatively associated with VAS (*B* = −0.187, *β* = −0.113, 95% CI: −0.316∼−0.058, *p* = 0.005); the model explained 40.8% of the variance. The VIF value is approximately 1.1, which is below 5, indicating that the estimated value is not significantly affected by multicollinearity. Component‐plus‐residual plots indicated an approximately linear relationship with a very small quadratic component for hardness that did not alter the conclusions (Table 2).

**TABLE 2:** Multiple linear regression of the association between upper trapezius muscle status and pain intensity (VAS).

Model	Variable	*B*	SE	*β*	95% CI	*p*	VIF
Model 1 *R* ^2^ = 0.188 Adjusted *R* ^2^ = 0.184	Mean hardness	0.085	0.012	0.324	0.062∼0.109	< 0.001^∗∗∗^	1.062
	Mean PPT	−0.364	0.075	−0.220	−0.510∼−0.217	< 0.001^∗∗∗^	1.062

Model 2 *R* ^2^ = 0.204 Adjusted *R* ^2^ = 0.194	Mean hardness	0.082	0.012	0.313	0.059∼0.106	< 0.001^∗∗∗^	1.073
	Mean PPT	−0.337	0.075	−0.204	−0.484∼−0.190	< 0.001^∗∗∗^	1.086

Model 3 *R* ^2^ = 0.423 Adjusted *R* ^2^ = 0.408	Mean hardness	0.071	0.010	0.268	0.051∼0.091	< 0.001^∗∗∗^	1.091
	Mean PPT	−0.187	0.066	−0.113	−0.316∼−0.058	0.005^∗∗^	1.135

*Note:* Model 1, unadjusted; Model 2, adjusted for age, sex, and BMI; Model 3, adjusted for age, sex, BMI, marital status, education level, type of work, smoking status, alcohol consumption, and daily working hours. *B*, unstandardized regression coefficient; *β*, standardized regression coefficient; *R* ^2^, coefficient of determination; adjusted *R* ^2^, coefficient of determination adjusted for the number of predictors.

Abbreviations: CI, confidence interval; Mean PPT, mean pressure pain threshold; SE, standard error; VIF, variance inflation factor.

^∗^ *p* < 0.05.

^∗∗^ *p* < 0.01.

^∗∗∗^ *p* < 0.001.

### 3.4. Regression Analysis of Upper Trapezius Status and Neck Disability

Regression analysis with NDI as the dependent variable showed that in the unadjusted model, mean hardness was significantly and positively associated with NDI (*β* = 0.193, *p* < 0.001), whereas mean PPT was not significantly associated with NDI (*β* = −0.074, *p* = 0.133). After full adjustment for covariates (Model 3), mean hardness retained a positive association with NDI (*B* = 0.180, *β* = 0.160, 95% CI: 0.075∼0.286, *p* < 0.001), and the association for mean PPT remained nonsignificant (*B* = −0.238, *β* = −0.034, 95% CI: −0.911∼0.435, *p* = 0.487); the adjusted model *R* ^2^ was 0.062 (Table 3).

**TABLE 3:** Multiple linear regression of the association between upper trapezius muscle status and neck disability (NDI).

Model	Variable	*B*	SE	*β*	95% CI	*p*	VIF
Model 1 *R* ^2^ = 0.050 Adjusted *R* ^2^ = 0.045	Mean hardness	0.218	0.055	0.193	0.110∼0.326	< 0.001^∗∗∗^	1.062
	Mean PPT	−0.520	0.345	−0.074	−1.198∼0.158	0.133	1.062

Model 2 *R* ^2^ = 0.067 Adjusted *R* ^2^ = 0.056	Mean hardness	0.209	0.055	0.185	0.101∼0.317	< 0.001^∗∗∗^	1.073
	Mean PPT	−0.395	0.347	−0.056	−1.078∼0.288	0.256	1.086

Model 3 *R* ^2^ = 0.086 Adjusted *R* ^2^ = 0.062	Mean hardness	0.180	0.054	0.160	0.075∼0.286	< 0.001^∗∗∗^	1.091
	Mean PPT	−0.238	0.342	−0.034	−0.911∼0.435	0.487	1.135

*Note:* Model 1, unadjusted; Model 2, adjusted for age, sex, and BMI; Model 3, adjusted for age, sex, BMI, marital status, education level, type of work, smoking status, alcohol consumption, and daily working hours. *B*, unstandardized regression coefficient; *β*, standardized regression coefficient; *R* ^2^, coefficient of determination; adjusted *R* ^2^, coefficient of determination adjusted for the number of predictors.

Abbreviations: CI, confidence interval; Mean PPT, mean pressure pain threshold; SE, standard error; VIF, variance inflation factor.

^∗^ *p* < 0.05.

^∗∗^ *p* < 0.01.

^∗∗∗^ *p* < 0.001.

### 3.5. Left–Right Side Analysis

When left and right muscle indicators were entered simultaneously into the fully adjusted model, for VAS right upper trapezius hardness (*B* = 0.053, 95% CI: 0.038∼0.068, *p* < 0.001) was more strongly associated with pain than left hardness (*B* = 0.018, 95% CI: 0.003∼0.033, *p* = 0.020), whereas neither left nor right PPT reached statistical significance (left: *B* = −0.080, 95% CI: −0.176∼0.016, *p* = 0.103; right: *B* = −0.081, 95% CI: −0.176∼0.015, *p* = 0.097). For NDI, only left upper trapezius hardness retained a significant association (*B* = 0.149, 95% CI: 0.068∼0.230, *p* < 0.001); right‐sided hardness and bilateral PPT were not significantly associated.

### 3.6. Subgroup Analyses

Prespecified subgroup analyses were conducted across nine variables: sex, age, BMI, marital status, education level, type of work, daily working hours, smoking status, and alcohol consumption (Figures 5 and 6). For VAS, mean hardness was positively associated with pain in all strata (all *p* < 0.05), and mean PPT was negatively associated with pain in nearly all strata. No significant interactions were observed (all *p* for interaction > 0.05). For NDI, the hardness coefficients were positive in nearly all strata and statistically significant in most of them; the exception was physical workers, among whom the coefficient was negative and nonsignificant (*B* = −0.122, 95% CI: −0.439∼0.195, *p* = 0.454), whereas the PPT association was generally nonsignificant. The smallest *p* value was for the hardness‐by‐work‐type interaction on the NDI (*p* for interaction = 0.122), which did not reach statistical significance; formal interaction tests therefore revealed no evidence of effect modification for either outcome. Differences in statistical significance across strata for either outcome did not reach significance.

**FIGURE 5:**
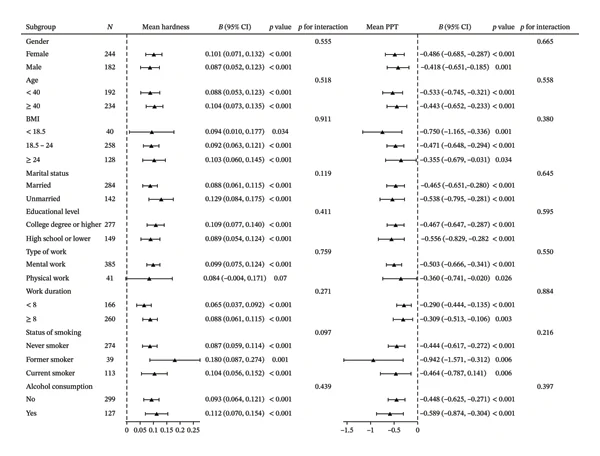
Forest plot of prespecified subgroup analyses for the associations of upper trapezius tissue hardness and pressure pain threshold with VAS score. B, unstandardized regression coefficient; CI, confidence interval.

**FIGURE 6:**
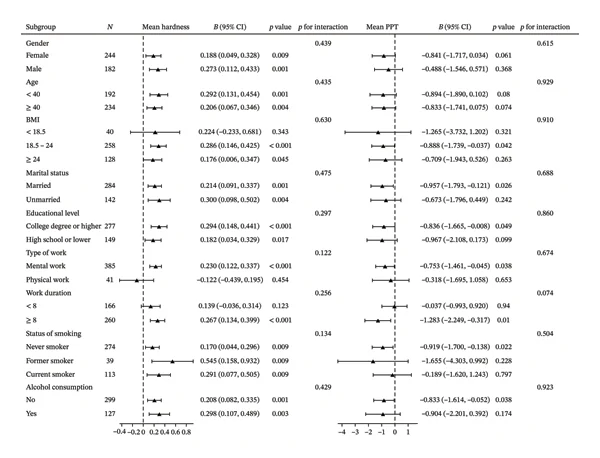
Forest plot of prespecified subgroup analyses for the associations of upper trapezius tissue hardness and pressure pain threshold with NDI score. B, unstandardized regression coefficient; CI, confidence interval.

## 4. Discussion

This study investigated the associations of upper trapezius tissue hardness and PPT with pain intensity and cervical dysfunction in 426 patients with CNSNP. The main findings are as follows: (1) both increased tissue hardness and decreased PPT were associated with pain intensity, and the standardized coefficient for hardness (*β* = 0.268) was larger than that for PPT (*β* = −0.113); (2) tissue hardness was also associated with cervical dysfunction, whereas PPT showed no independent association with functional impairment; (3) the side‐specific patterns differed between outcomes, with right‐sided hardness more strongly associated with pain and left‐sided hardness with disability; and (4) formal interaction tests indicated that these associations were generally consistent across the demographic and clinical characteristics examined.

### 4.1. Associations of Upper Trapezius Tissue Hardness With Pain Intensity and Neck Disability

We found that upper trapezius tissue hardness was weakly to moderately positively correlated with VAS scores (*r* = 0.328), and this association persisted after comprehensive adjustment (*β* = 0.268), which is broadly consistent with prior literature using related measurement techniques. Itoh et al. reported a significant correlation between upper trapezius hardness and VAS scores in patients with neck pain (*r* = 0.41) [28]. Castaldo et al. found that upper trapezius mechanical measures on the affected side were higher in patients with neck pain than in controls and were positively correlated with pain intensity [13]. Because indentation tissue hardness and shear‐wave elastography quantify different physical quantities, these convergent findings are supportive rather than directly interchangeable. Several mechanisms could plausibly link greater tissue hardness to pain. Increased hardness may reflect changes such as sarcomere shortening, altered calcium regulation, or extracellular matrix fibrosis that can activate or sensitize intramuscular nociceptors [29, 30]. It may also be accompanied by reduced local perfusion and accumulation of metabolic products [31], and it can alter the biomechanical loading of cervical segments, facet joints, and intervertebral discs [32]. These pathways remain hypothetical in the present cross‐sectional data.

Upper trapezius tissue hardness was also positively associated with NDI scores (*β* = 0.160, *p* < 0.001), although more weakly than with VAS. Hardness could relate to daily function not only through pain but also by restricting cervical range of motion and altering motor control, and Taş et al. reported associations between muscle stiffness and cervical range of motion and functional status [8]. The explanatory power of the NDI model was low (adjusted *R* ^2^ = 0.062), in contrast to the VAS model (adjusted *R* ^2^ = 0.408), indicating that disability is shaped by many determinants not captured here. Beyond muscle status, psychological factors such as fear‐avoidance beliefs, anxiety, and depression, together with social factors and altered central pain processing, may be more important for disability [33, 34], and these constructs were not measured in the present study.

### 4.2. The Association Between PPT, Pain Intensity, and Neck Disability

We found that upper trapezius PPT was significantly and negatively associated with VAS scores (*r* = −0.307), and this association remained in the fully adjusted model (*β* = −0.113), whereas PPT was not significantly associated with NDI. A lower PPT at the symptomatic upper trapezius indicates greater local pressure pain sensitivity, that is, mechanical hyperalgesia at the measurement site [35]. Such local hyperalgesia can arise through peripheral sensitization and, in some patients, may be accompanied by central sensitization; central sensitization is therefore one possible interpretation rather than something the present PPT measurement establishes directly. Importantly, we measured PPT only over the symptomatic upper trapezius and did not assess distant, asymptomatic sites or include other measures of nociceptive processing, so we cannot determine whether pain hypersensitivity was widespread, which is the hallmark of central sensitization [36, 37]. The smaller coefficient for PPT than for hardness may reflect the fact that the composite VAS score combines current and recalled pain over the previous 4 weeks, whereas PPT characterizes local pressure pain sensitivity at the time of testing; the two are related but not equivalent.

The lack of an independent association between PPT and NDI is consistent with the broader literature. A recent systematic review of predictors of persistent and recurrent nonspecific neck pain concluded that the evidence for an association between cervical PPT and persistent disability was inconsistent [38]. This may reflect the complexity of disability, which spans physiological, psychological, and social dimensions and is unlikely to be captured by a single local measure of pain sensitivity.

### 4.3. Left–Right Differences and Sex Differences

We found that tissue hardness was generally higher on the right than on the left and that right‐sided hardness showed a stronger association with VAS. Because handedness was not assessed in this study, the possible contribution of dominant‐side overuse, suggested by ergonomic studies of upper‐limb asymmetry during repetitive work [39, 40], remains speculative. Left‐sided hardness was more strongly associated with disability in the side‐specific model. This difference between outcomes was not anticipated and may reflect chance or multiple testing rather than a genuine lateralized mechanism; it could also relate to central integration of bilateral sensory input, but this is conjectural. Longitudinal studies that assess handedness and bilateral function are needed to clarify these findings.

Female patients in this study had higher VAS and NDI scores and significantly lower PPT than male patients, consistent with previous epidemiological observations of higher neck pain prevalence and greater pain severity in women [41]. Greater pain sensitivity in women may be related to sex hormone levels, differences in endogenous opioid system function, and psychosocial factors [42]. Despite these baseline differences, subgroup analyses showed no significant interaction between sex and the associations of tissue hardness and PPT with clinical outcomes, suggesting that these associations are consistent across sexes.

### 4.4. Clinical Implications

These findings suggest several considerations for clinical practice and future research. First, upper trapezius tissue hardness and PPT may provide additional objective information that complements subjective scales such as VAS and NDI, but this cross‐sectional study does not establish their incremental clinical value beyond conventional assessment, particularly given the limited explanatory power of the disability model (adjusted *R* ^2^ = 0.062). Second, because hardness was associated with both pain and disability, it is a candidate marker for prospective evaluation; whether reducing hardness through manual therapy, dry needling, muscle energy techniques, or stretching actually improves pain and function should be tested in controlled intervention studies rather than inferred from these cross‐sectional data. Third, a low local PPT identifies greater local pressure pain sensitivity; whether such patients benefit from strategies that address central sensitization requires studies that include widespread pain testing. Fourth, the observed side differences suggest that bilateral assessment is reasonable, but they do not establish one side as a treatment priority.

### 4.5. Limitations

This study has several limitations. First, the cross‐sectional design precludes causal inference, and longitudinal or interventional studies are needed to establish the direction of the relationships. Second, eligibility was conditioned on VAS ≥ 3 and NDI ≥ 10. Selecting participants on the basis of the outcomes truncates their distributions and may alter the estimated strength of the associations; the results therefore should not be generalized to patients across the full spectrum of CNSNP, including those with milder symptoms. Third, as a single‐center sample that was predominantly composed of mental workers (90.4%), the findings may not extend to other occupational settings or populations. Fourth, psychosocial factors that influence neck pain and disability, including fear‐avoidance beliefs, anxiety, and depression, were not measured. Fifth, the OE220 provides a relative indentation hardness value rather than an absolute elastic modulus, and its output should not be equated directly with shear‐wave elastography. Sixth, the specific composite pain score has not been independently validated as a single equally weighted outcome, and it could not be decomposed in a sensitivity analysis because the three individual VAS ratings were not retained. Finally, electromyography was not performed, so the interplay among muscle activation, hardness, and pain remains unexplored.

## 5. Conclusions

This cross‐sectional study found that in patients with CNSNP, increased upper trapezius tissue hardness and decreased local PPT were associated with pain intensity, and that hardness was also associated with neck disability. The associations did not differ significantly across the examined subgroups (all *p* for interaction > 0.05; hardness by type of work for the NDI, *p* for interaction = 0.122), providing no statistically significant evidence of effect modification. These findings suggest that objective upper trapezius measures may provide complementary descriptive information in the assessment of CNSNP, although their incremental clinical value has not been established. Whether these measures can guide treatment decisions or predict treatment response remains a question for future longitudinal and interventional research.

## Funding

This study was partly supported by the National Natural Science Foundation of China (82575252); the Science and Technology Development Project of Shanghai University of Traditional Chinese Medicine in 2024 (24KFL033); the Seed Funding Program for Outstanding Young Researchers, Shanghai University of Traditional Chinese Medicine (26YTJ XLZ03); the Inheritance Program of Haipai Traditional Chinese Medicine for Young and Middle‐aged Physicians (2026‐HPZY‐01); and the Shanghai Municipal Public Health Research Special Project (2025GKQ14).

## Conflicts of Interest

The authors declare no conflicts of interest.

## Data Availability

The data that support the findings of this study are available from the corresponding author upon reasonable request.
